# Formation and Abundance of 5-Hydroxymethylcytosine in RNA

**DOI:** 10.1002/cbic.201500013

**Published:** 2015-02-12

**Authors:** Sabrina M Huber, Pieter van Delft, Lee Mendil, Martin Bachman, Katherine Smollett, Finn Werner, Eric A Miska, Shankar Balasubramanian

**Affiliations:** [a] University of Cambridge, Department of ChemistryLensfield Road, Cambridge, CB2 1EW (UK); [b] Cancer Research UK Cambridge Institute, University of CambridgeLi Ka Shing Centre, Robinson Way, Cambridge, CB2 0RE (UK); [c] University College London, Institute for Structural and Molecular BiologyGower Street, London, WC1E 6BT (UK); [d] University of Cambridge, Wellcome Trust Cancer Research UK Gurdon InstituteTennis Court Road, Cambridge, CB2 1QN (UK)

**Keywords:** 5-hydroxymethylcytosine, 5-methylcytosine, isotope tracing, LC-MS/MS, RNA modifications

## Abstract

RNA methylation is emerging as a regulatory RNA modification that could have important roles in the control and coordination of gene transcription and protein translation. Herein, we describe an in vivo isotope-tracing methodology to demonstrate that the ribonucleoside 5-methylcytidine (m^5^C) is subject to oxidative processing in mammals, forming 5-hydroxymethylcytidine (hm^5^C) and 5-formylcytidine (f^5^C). Furthermore, we have identified hm^5^C in total RNA from all three domains of life and in polyA-enriched RNA fractions from mammalian cells. This suggests m^5^C oxidation is a conserved process that could have critical regulatory functions inside cells.

Half a century of research has identified more than 140 distinct post-transcriptional modifications of ribonucleic acids.[1] The majority of these chemical modifications are found in the highly abundant transfer and ribosomal RNAs (tRNA, rRNA, 95 % of total RNA) where they contribute to RNA folding, provide structural stability and diversify molecular recognition, for example, through “wobble base pairing”.[2] Recently RNA modifications have also been identified in the less abundant messenger and long noncoding RNAs (mRNA, lncRNA).[3, 4] Given the short half-life of many RNA species (*t*_1/2_<10 h), RNA modifications previously have been viewed largely as static and stable marks. Recent studies, however, have shown that they can be dynamic and might have critical regulatory and/or signalling roles inside cells, similar to the reversible epigenetic marks on DNA and histones.[5] For instance, *N*^*6*^-methyladenosine (m^6^A), the most abundant internal modification in eukaryotic mRNA, undergoes oxidative processing to *N*^*6*^-hydroxymethyladenosine and *N*^*6*^-formyladenosine by the fat mass and obesity-associated protein (FTO) and ALKBH5, respectively.[6, 7] Although the function of this dynamic adenosine methylation is not yet fully understood, it is proposed to be involved in the regulation of gene expression, mRNA stability and protein translation.[8, 9] 5-Methylcytidine (m^5^C) is another known methylated ribonucleoside that is widespread across both coding and noncoding RNAs from all domains of life.[2] Although 5-methyl-2′-deoxycytidine (m^5^dC) is known to be an inheritable epigenetic mark in DNA; the precise cellular and molecular functions of m^5^C-modified nucleobases in RNA are not clearly understood. Recent studies have shown that m^5^C sites in lncRNAs can affect binding to chromatin-modifying complexes in vitro;[4] this implies that RNA C5-methylation can affect the epigenetic status of a cell. Furthermore, m^5^C in vault RNA has been demonstrated to act as a molecular switch to produce specific small vault RNAs, which can enter the micro RNA pathway and influence the translation of distinct sets of mRNAs.[10] These findings provide the first evidence that m^5^C in RNA could be critical for the control and regulation of gene transcription and protein translation, and prompted us to investigate whether m^5^C in RNA is subject to dynamic control inside organisms in a fashion similar to m^6^A.

Subsequent to the recent discovery of 5-hydroxymethyl-2′-deoxycytosine (hm^5^dC) in mammalian DNA,[11] the dynamics and oxidative metabolism of DNA C5-methylation have been the subject of intense investigation. The ten-eleven translocation (TET) family of enzymes can mediate the removal of m^5^dC through an oxidative pathway involving the distinct intermediates hm^5^dC, 5-formyl-2′-deoxycytosine and 5-carboxy-2′-deoxycytosine.[12] Furthermore, studies on the lifetime of hm^5^dC have demonstrated that it can be a stable DNA modification, thus suggesting that hm^5^dC might have its own unique biological function in addition to being an intermediate in m^5^dC removal.[13]

In RNA, hm^5^C was originally identified in rRNA isolated from wheat seedlings.[14] During the preparation of this manuscript, Fu et al. reported the presence of hm^5^C for the first time in mammalian total RNA and demonstrated that it can be formed in vitro by TET-mediated oxidation of m^5^C.[15]

Herein, we present direct chemical evidence for the in vivo oxidative processing pathway of m^5^C in RNA. Using an isotope-tracing strategy in a mouse model followed by liquid chromatography tandem-mass spectrometry (LC-MS/MS) analysis, we were able to follow m^5^C metabolism in RNA. Furthermore, we quantified hm^5^C in total RNA samples from all domains of life and polyA-enriched mammalian RNA to better understand the scope and biological function of this noncanonical base in RNA.

To explore the in vivo metabolic pathway of 5-methylcytosine in RNA and determine whether hm^5^C is indeed the result of m^5^C oxidation, we carried out an isotope-tracing experiment by using a mouse model. RNA methyltransferases (RMTases) use *S*-adenosylmethionine (SAM) as the methyl-group donor in all nucleoside methylation events.[2] As SAM is synthesized from l-methionine,[16] feeding mice on a diet supplemented with ^13^C and D stable-isotope-labelled methionine leads to the incorporation of these isotopes into the C5-methyl group of m^5^C. Consequently, isolation of the murine RNA and the LC-MS/MS analysis thereof, enables the detection of isotope-labelled m^5^C and any derivatives that are formed directly from it.

Mice were fed on a custom diet depleted of l-methionine and supplemented with l-[methyl-^13^CD_3_]methionine for 117 days.[13] The mice were then sacrificed, and RNA was extracted from their brain tissue, which was then digested by a mixture of nucleases and phosphatases to form the nucleoside monomers. Incorporation of the ^13^CD_3_ label was then assessed by LC-MS/MS analysis of the RNA nucleosides, with the mass filters of the triple quadrupole preset for m^5^C and m^5^C+4 Da. This revealed that the ^13^CD_3_ label had been incorporated in 51 % of total m^5^C (Scheme 1). Next, we detected the masses of hm^5^C and f^5^C and their expected isotopologues hm^5^C+3 Da and f^5^C+2 Da, respectively. As depicted in Scheme1, we observed ^13^C and D isotope incorporation into hm^5^C and f^5^C with similar efficiencies as for m^5^C (hm^5^C: 41 %, f^5^C: 39 %). These findings confirm that m^5^C in RNA can be oxidatively metabolized first to hm^5^C and subsequently to f^5^C. In the recently published study on TET-mediated oxidation of m^5^C to hm^5^C in synthetic RNA oligonucleotides, no significant sequential in vitro oxidation of hm^5^C to f^5^C was observed.[15]

**Scheme 1:**
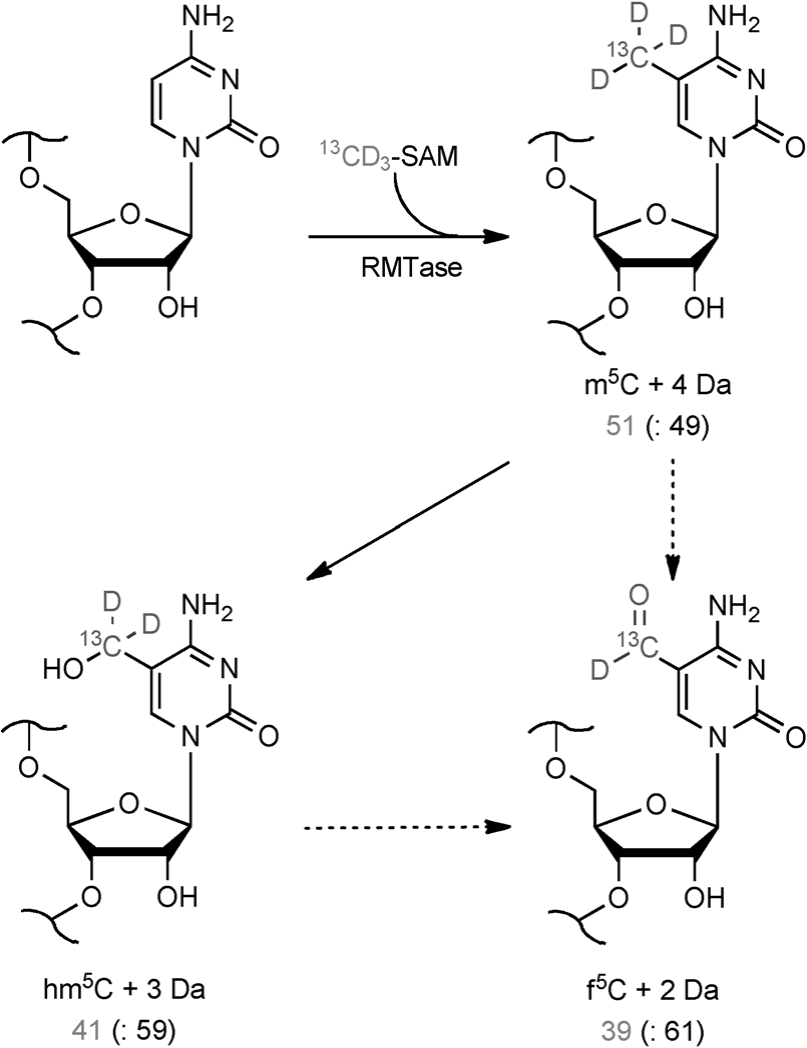
In vivo methylation and subsequent oxidation of cytidine as detected by MS analysis of RNA from the brain of mice fed with l-[methyl-^13^CD_3_]methionine. Numbers correspond to the ratio of peak areas of the depicted labelled nucleosides (in grey) against the respective unlabelled nucleosides (in brackets). See Figure S6.1 and Table S6.3 for extracted ion count chromatograms and absolute values obtained thereof, and Table S6.4 for HR-LC-MS/MS data.

The conservation of m^5^C in RNA across Archaea, bacteria and eukaryotes[2] and the apparent metabolic relationship between m^5^C and hm^5^C in vivo suggest that RNA C5-hydroxymethylation could be a widespread process. To asses the prevalence of hm^5^C across different species, a quantitative LC-MS/MS method was implemented with stable isotope-labelled (SIL) internal standards, which were spiked into samples and calibration standards.[17] Its presence and concentrations were determined by using both the LC and MS/MS selectivity. To apply this method to the detection and quantification of m^5^C and hm^5^C in RNA, SIL standards for cytidine, m^5^C and hm^5^C were synthesised as depicted in Scheme 2.[18] Thus, 2-^13^C,^15^N_2_-labelled uracil was synthesized from a commercial source of [2-^13^C,^15^N_2_]urea and used in a Vorbrüggen coupling with an acyl-protected ribose donor in the presence of a Lewis acid to yield 2′,3′,5′-tri-*O*-benzoyl-[2-^13^C,1,3-^15^N_2_]uridine (**2**; Scheme 2 A). Treatment of **2** with tri-(1,2,4-triazol-4-yl)-*N*-phosphoramidate furnished an intermediate N4-triazolyl species. Subsequent aminolysis with concentrated aqueous NH_4_OH and saponification of the benzoyl esters under Zemplén conditions yielded [2-^13^C,1,3-^15^N_2_]cytidine (**3**). 5-Hydroxymethyl-[2-^13^C,1,3-^15^N_2_]cytidine (**4**) was obtained from hydroxymethylation of **3** by adapting published procedures in a microwave-assisted formylation reaction in combination with the use of a cyclic acetal protecting group to aid purification.[19] 5-[Methyl-D_3_]-[6D]cytidine (**6**) was synthesized from [D_4_]thymine by using the same sequence of reactions as was used for the synthesis of **3** (Scheme 2 B).

**Scheme 2:**
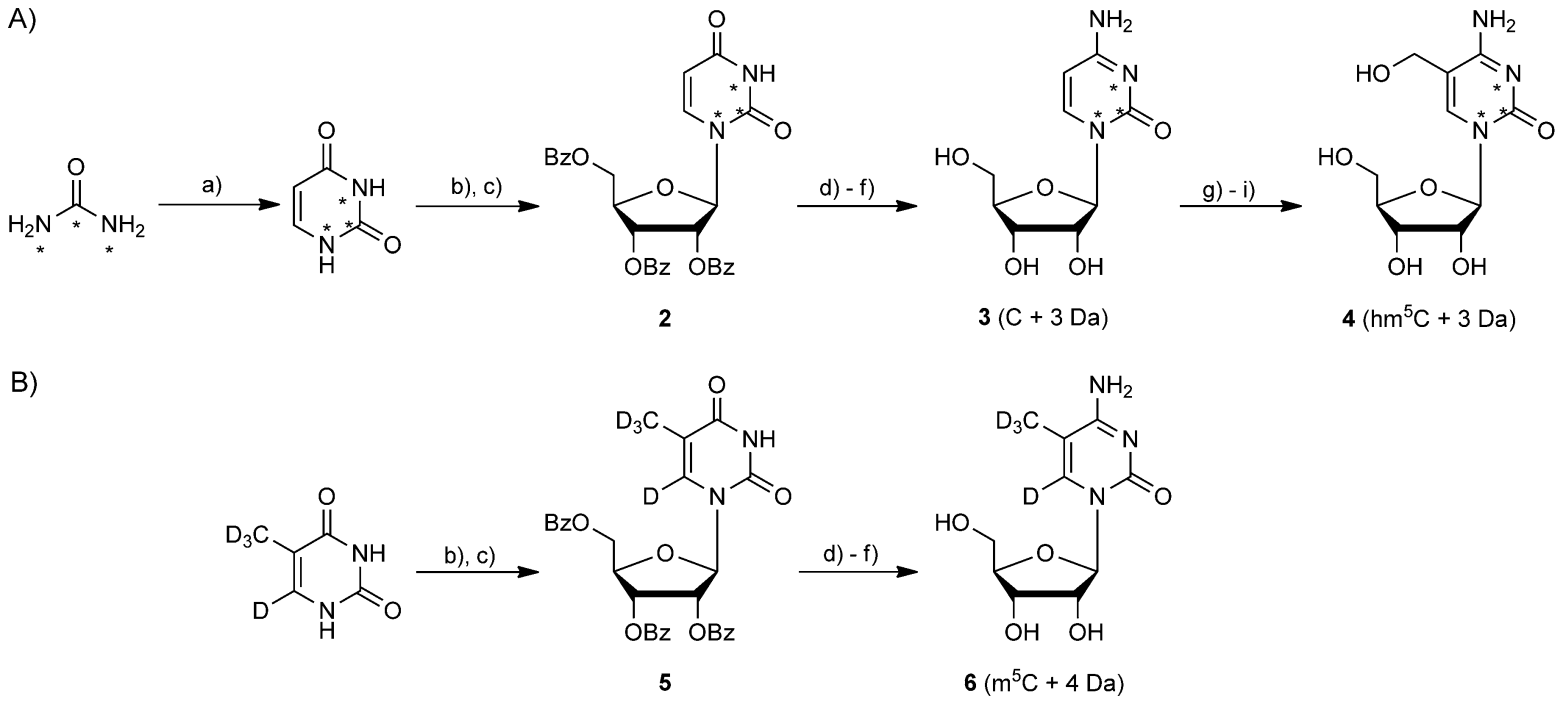
Synthesis of stable-isotope-labelled cytidine, 5-methylcytidine and 5-hydroxymethylcytidine: a) polyphosphoric acid, 17 h, 95 °C, 37 %; b) TMSCl, 1,1,1,3,3,3-hexamethyldisilazane, MeCN, 14 h, 85 °C; c) 1-*O*-acetyl-2,3,5-tri-*O*-benzoyl-β-D-ribofuranose, TMSOTf, 1,2-dichloroethene, 4 h, 70 °C, 80 %; d) tris(3,5-dihydro-4*H*-1,2,4-triazol-4-yl)phosphine oxide, MeCN, 17 h, 25 °C; e) NH_4_OH (35 %), 1,4-dioxane, 2 h, 25 °C, 69 %; f) NaOMe, MeOH, 5 h, 25 °C, 97 %; g) H_2_SO_4_, acetone, 15 h, 25 °C, 87 %; h) paraformaldehyde, KOH (0.5 M), microwave irradiation, 75 min, 60 °C, 16 %; i) 90 % TFA, 1 h, 0 °C, 98 %. Asterisks represent a ^15^N or ^13^C isotopologue.[18]

With the stable-isotope-labelled internal standards in hand, we examined the abundance of hm^5^C in total RNA samples from model organisms representing the three domains of life by using a previously described methodology.[20] We chose the archaeal species *Methanocaldococcus jannaschii*, the bacterium *Escherichia coli* and two eukaryotic model organisms, namely a plant (*Arabidopsis thaliana*) and a nematode (*Caenorhabditis elegans*). HEK293T cells and mammalian tissues[21] were analysed to verify previously reported results.[15] To rule out oxidation of m^5^C to hm^5^C as a result of downstream RNA processing, we subjected synthetic RNA 122-mers to identical RNA extraction and enzymatic digestion. No oxidation was detected in the quantitative LC-MS/MS analysis of these control samples.

We detected hm^5^C in all samples studied (Figure 1), thus confirming RNA C5-hydroxymethylation as a common RNA modification. Notably, hm^5^C was found in species that do not exhibit detectable hm^5^dC in their DNA and lack TET homologues in their genomes, such as *C. elegans* and *A. thaliana.*[22] This suggests that, in such organisms, the formation of hm^5^C in RNA must occur through a non-TET mechanism. As noted by Fu et al., their observation that TET-null ES cells in which TET1, TET2, and TET3 are genetically deleted still exhibit significant hm^5^C levels in RNA also supports the idea that hm^5^C in RNA can form from pathways that do not involve TET enzymes.[15] Comparison of m^5^C oxidation into hm^5^C across the different species revealed varying levels (Figure 2). This could also indicate that RNA hydroxymethylation is the result of different enzymatic transformations. It will ultimately be essential to map the position of hm^5^C to specific RNAs in order to understand its function. As a step towards elucidating hm^5^C location, we explored the abundance of hm^5^C in RNA classes other than tRNA and rRNA. Specifically, we isolated mRNA and lncRNAs from HEK293T cells that contain a polyA tail by RNA pulldown using polyT magnetic beads. Subsequent analysis of this fraction by LC-MS/MS allowed us to measure hm^5^C (Figure S6.2 in the Supporting Information). In these samples, the extent of C5-hydroxymethylation in polyA RNA was 40 times higher than in total RNA (Table S6.5). Currently, the function of m^5^C in mRNA and lncRNA is not clearly understood. However, the presence and levels of hm^5^C as an oxidative m^5^C metabolite in polyA RNA suggests that hm^5^C and m^5^C might be part of a dynamic regulatory mechanism. Further studies are needed to assess the functional roles of m^5^C and hm^5^C in RNA.

**Figure 1:**
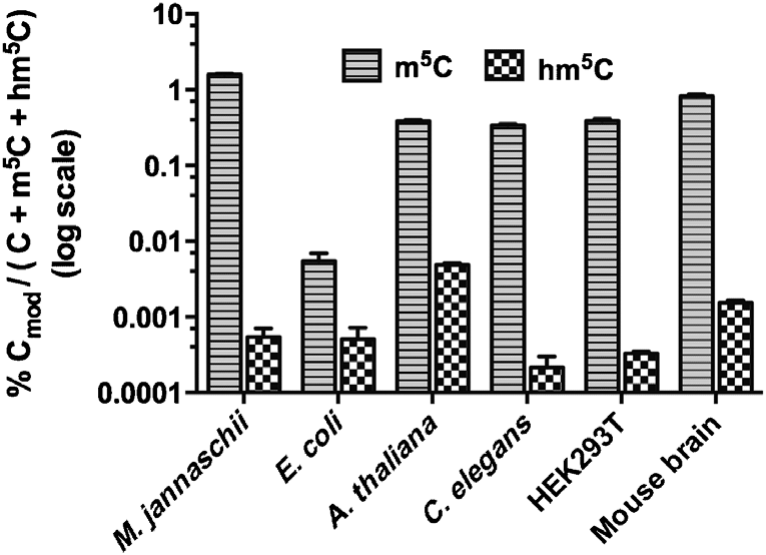
Levels of m^5^C and hm^5^C across different model organisms given in amounts relative to the sum of (modified) cytosine residues.[20]

**Figure 2:**
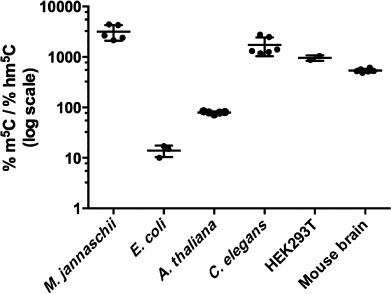
The abundance of hm^5^C as a fraction of m^5^C in various organisms.

In summary, we have shown for the first time that 5-hydroxymethylcytosine in RNA is generated by m^5^C oxidation in vivo and is pervasive in all three domains of life across a variety of different species. Our findings suggest the existence of dynamic m^5^C metabolism in RNA that might have important roles in RNA function.

## Experimental Section

All in vivo experiments were performed under the terms of a UK Home Office licence. C57BL/6J (JAX mice strain) mice were bred and housed according to UK Home Office guidelines. Detailed information on the mouse labelling study, cell culture and subsequent RNA extraction (TRI reagent, Sigma) and purification (RNA clean & concentrator kit, Zymo Research*)* is given in the Supporting Information. Synthetic procedures and full compound characterization for all compounds and ^1^H and ^13^C NMR spectra of **3**, **4** and **6** can also be found in the Supporting Information.

RNA was digested by using a mixture of Benzonase (Sigma), snake venom phosphodiesterase I (*Crotalus adamanteus* venom, Sigma) and antarctic phosphatase (New England Biolabs) in a Tris**⋅**HCl buffer (pH 8) for 12 h. A solution of SIL C, m^5^C and hm^5^C used for LC-MS/MS quantitation was added to samples before the enzymatic digestion.

LC-MS/MS analysis was performed by using an Agilent 1290 Infinity UHPLC system coupled to an AB Sciex 6500 triple-quadrupole mass spectrometer. Chromatographic conditions typically consisted of using a Waters Acquity UPLC HSS T3 column and an H_2_O/MeCN (0.1 % formic acid) linear gradient.
