# Supplementary material for: Formation and Abundance of 5-Hydroxymethylcytosine in RNA
Source: Chembiochem. 2015 Feb 12;16(5):752–5. doi: 10.1002/cbic.201500013 (PMC4471624; doi:10.1002/cbic.201500013)

CHEMBIOCHEM

Supporting Information

Formation and Abundance of 5-Hydroxymethylcytosine in RNA

Sabrina M. Huber,[a] Pieter van Delft,[a] Lee Mendil,[b] Martin Bachman,[b] Katherine Smollett,[c] Finn Werner,[c] Eric A. Miska,[d] and Shankar Balasubramanian*[a, b]

cbic_201500013_sm_miscellaneous_information.pdf

Supporting Information

1. Materials: 2

2. Synthesis of stable isotope-labeled standards for LC-MS/MS analysis 3

2.1 [2-^{13}C, 1,3-$^{15}N_2$]-Uracil (**1**) 3

2.2 2',3',5'-Tri-O-benzoyl-[2-^{13}C, 1,3-$^{15}N_2$]-uridine (**2**) 3

2.3 Tri-(4H-1,2,4-triazol-4-yl)phosphine oxide 4

2.4 2',3',5'-Tri-O-benzoyl-[2-^{13}C, 1,3- $^{15}N_2$]-cytidine 4

2.5 [2-^{13}C, 1,3-$^{15}N_2$]-Cytidine (**3**) 5

2.6 [2-^{13}C, 1,3-$^{15}N_2$]-2',3'-O-isopropylidene cytidine 5

2.7 5-Hydroxymethyl-[2-^{13}C, 1,3-$^{15}N_2$]-2',3'-O-isopropylidene cytidine 6

2.8 5-Hydroxymethyl-[2-^{13}C, 1,3-$^{15}N_2$]-cytidine (**4**) 6

2.9 5-[Methyl-D_3]-[6-D]-cytidine (**6**) 6

3. Isolation of total RNA, purification and polyA pull-down 7

4. RNA digestion 7

5. LC-MS/MS analysis 7

5.1 LC-MS/MS analysis of labeled murine tissues 7

5.2 Quantitative LC-MS/MS analysis 8

6. Tables and Figures: 9

Table S6.1: The levels of hm^{5}C in total RNA samples isolated from different model organisms and various male mouse tissues 9

Table S6.2: The levels of m^{5}C in total RNA samples isolated from different model organisms and various male mouse tissues 9

Figure S6.1: Extracted ion count (XIC) for m^{5}C + 4 Da, hm^{5}C + 3 Da, f^{5}C + 2 Da and their unlabeled analogues 10

Table S6.3: A) Absolute XIC values for nucleosides measured in the SIL-mouse tissues B) Relative distribution of mouse tissue nucleosides derived from A. 11

Table S6.4: Accurate masses of nucleobases detected in RNA digests from SIL-mouse tissues. 11

Table S6.5: The levels of m^{5}C and hm^{5}C in polyA and total RNA samples isolated from HEK293T cells. 11

Figure S6.2: Extracted ion count (XIC) for hm^{5}C in polyA RNA from HEK293T cells. 12

Figure S6.3: hm^{5}C abundance measured in murine tissues, relative to the sum of rC-nucleosides. 12
Figure S6.4a: Representative calibration curve for C-detuned. 13
Figure S6.4b: Representative calibration curve for m^{5}C. 13
Figure S6.4c: Representative calibration curve for hm^{5}C. 13

7. NMR spectra of SILs 14
7.1 [2-^{13}C, 1,3-$^{15}N_2$]-Cytidine (**3**) 14
7.2 5-[Methyl-D3]-[6-D]-cytidine (**6**) 15
7.3 5-Hydroxymethyl-[2-^{13}C, 1,3-$^{15}N_2$]-cytidine (**4**) 16

1. Materials:

All solvents and reagents were purchased from *Sigma Aldrich*, *Alfa Aesar*, or *Fisher Scientific* and used as received. Solvents MeCN and toluene were dried by distillation over CaH_2. DCE and acetone were dried using flame dried 3 Ångstrom molecular sieves. Unless stated otherwise all reactions were performed at ambient temperature and under an argon atmosphere. TLC was performed on ALUGRAM SIL G/UV254 (*Macherey-Nagel*) pre-coated TLC sheets. Flash chromatography was carried out using CombiFlash Rf (Teledyne Isco) with puriFlash columns (*Interchim*). Microwave reactions were performed using a Discover SP (CEM) system. ^{1}H-NMR and ^{13}C-NMR spectra were recorded on a Bruker DRX-400, Bruker DPX-400 or Bruker DRX-500 instrument and are referenced to the residual solvent peak. Chemical shifts are quoted in parts per million (ppm) using the following abbreviations: s, singlet; d, doublet; t, triplet; m, multiplet; br, broad. The coupling constants (*J*) are measured in Hertz.

Mouse tissues used for the quantification of m^{5}C and hm^{5}C were from 62 days old C57BL/6J (JAX mice strain) male mice. Isotope-labelled brain tissues were obtained from male and female C57BL/6J (JAX mice strain) mice that had been fed with a custom *L*-methionine-free mouse diet supplemented with *L*-[*methyl*-$^{13}CD_3$]-methionine (*Sigma Aldrich*) that was manufactured by TestDiet for 117 days. *A. thaliana* total RNA was obtained from Prof. Baulcombe (*University of Cambridge*) and *M. jannaschii* total RNA was provided by Prof Werner (*University College London*).

2. Synthesis of stable isotope-labeled standards for LC-MS/MS analysis

2.1 [2-^{13}C, 1,3-$^{15}N_2$]-Uracil (1)

Polyphosphoric acid (27 g, 79.9 mmol) was stirred at 120 °C for 20 min before [^{13}C,$^{15}N_2$]-urea (950 mg, 15.08 mmol) was added at 95 °C. Upon formation of a homogeneous reaction mixture, propiolic acid (1.2 ml, 19.5 mmol) was added and the solution was stirred at 95 °C for 17 h. The reaction mixture was diluted with H_2O (40 ml) and aqueous NH_4OH (35 %, 35 ml) to increase the pH to 7. followed by the evaporation of the solvent under reduced pressure. The residue was dissolved in aqueous NH_4OH (35 %, 125 ml), MeOH (125 ml) was added and the reaction mixture was stirred at 25 °C for 2 h. The colourless salt was removed by vacuum filtration and washed with aqueous NH_4OH/MeOH 1:1. The filtrate was concentrated under reduced pressure yielding the title compound. [2-^{13}C, 1,3-$^{15}N_2$]-Uracil (**1**) was obtained after recrystallization from H_2O as a pale yellow solid (640 mg, 5.57 mmol, 37 %). ^{1}H-NMR (DMSO-*d*^{6}, 400 MHz): δ 11.00 (*s*, 1 H, H3) 10.80 (*s*, 1 H, H1), 7.38 (*d*, *J* = 7.6 Hz, 1 H, H6), 5.44 (*d*, *J* = 7.7 Hz, 1 H, H5). ^{13}C-NMR (DMSO-*d*^{6}, 100 MHz): δ 164.4 (C4), 151.5 (C2), 142.2 (C6), 100.3 (C5). HRMS (ESI$^+$): calc. for $C_3{}^{13}CH_4{}^{15}N_2O_4$ $[M+H]^+$: 116.0320, found: 116.0319.

2.2 2',3',5'-Tri-O-benzoyl-[2-^{13}C, 1,3-$^{15}N_2$]-uridine (2)

1-*O*-Acetyl-2,3,5-tri-*O*-benzoyl-β-*D*-ribofuranose (2190 mg, 4.34 mmol) and [2-^{13}C, 1,3-$^{15}N_2$]-uracil (580 mg, 1.16 eq) were co-evaporated twice with dry acetonitrile (MeCN). MeCN (30 ml), hexamethyldisilazane (HMDS) (1.1 ml, 1.2 eq) and trimethylsilyl chloride (TMS-Cl) (661 µl, 1.2 eq) were added and the reaction mixture was refluxed for 14 h. Solvents were removed under reduced pressure and the residue was co-evaporated twice with dry toluene. The oily residue was redissolved in 1,2-dichloroethane (DCE) (22 ml), TMS-OTf in DCE (0.5 M, 8.7 ml) was added drop wise at 0 °C followed by refluxing at 70 °C for 4 h. The reaction mixture was quenched with saturated aqueous $NaHCO_3$ and the aqueous phase was extracted with dichloromethane (DCM). The organic phases were washed with brine and dried over $MgSO_4$. Silicagel flash column chromatography (EtOAc : Hexane, 10 : 90 → 50 :50, v/v) yielded the title ribonucleoside 2',3',5'-tri-*O*-benzoyl-[2-^{13}C, 1,3-$^{15}N_2$]uridine (**2**) as a colourless foam (1.94 g, 3.47 mmol, 80 %). ^{1}H-NMR (DMSO-*d*^{6}, 400 MHz): δ 11.49 (*s*, 1 H, NH), 7.99 (*m*, 2 H, Ph-H), 7.87 (*m*, 4 H, Ph-H), 7.82 (*d*, *J* = 8.0 Hz, 1 H, H6), 7.63 (*m*, 3 H, Ph-H), 7.49 (*m*, 2 H, Ph-H), 7.43 (*m*, 4 H, Ph-H), 6.14 (*m*, 1 H, H1'), 5.91 (*m*, 2 H), 5.66 (*d*, *J* = 8.0 Hz, 1 H, H5), 4.67 (*m*, 3 H). ^{13}C-NMR (DMSO-*d*^{6}, 100 MHz): δ 165.5, 164.7, 163.2, 150.4, 142.4, 134.0, 133.9, 133.6, 129.4, 129.3, 128.8, 128.6, 128.5, 102.3, 89.7, 79.2, 78.8, 73.3, 70.6, 63.7. HRMS (ESI$^+$): calc. for $C_{29}{}^{13}CH_{24}{}^{15}N_2O_9$ $[M+H]^+$: 560.1529, found: 560.1527.

*2.3 Tri-(4*H*-1,2,4-triazol-4-yl)phosphine oxide*

To a vigorously stirred suspension of 1,2,4-triazole (2.28 g, 62 mmol) in anhydrous MeCN (56 ml) at -15 °C was added dropwise $POCl_3$ (0.7 ml, 7.6 mmol) followed by the dropwise addition of triethylamine (TEA) (4.96 ml, 35.5 mmol). The resulting thick slurry was stirred at -5 °C for 1 h. The colourless precipitate was allowed to settle and the supernatant, containing the title tri-*N*-triazolylphosphoramidate, was stored as a solution in MeCN at 4 °C and used directly in the next step.

2.4 2',3',5'-Tri-O-benzoyl-[2-^{13}C, 1,3- $^{15}N_2$]-cytidine

2',3',5'-Tri-*O*-benzoyl-[2-^{13}C, 1,3-$^{15}N_2$]uridine (**2**) (1.0 g, 1.79 mmol) was co-evaporated twice with dry MeCN (from 2.3) and redissolved in MeCN (24 ml). Tri-(4*H*-1,2,4-triazol-4-yl)phosphine oxide in MeCN (15 ml) was added and the reaction mixture was stirred at room temperature for 17 h. The yellow reaction mixture was quenched by the addition of TEA (1.48 ml) and H_2O (2 ml) and stirred at room temperature for 5 min. The reaction mixture was diluted with EtOAc and extracted with aqueous $NaHCO_3$ and brine. The organic phase was dried over $MgSO_4$ and the solvent was removed under reduced pressure to yield a bright yellow oil. The crude oil was taken up in 1,4-dioxane (33 ml) and concentrated NH_4OH (35 %, 10 ml) was added. The reaction mixture was stirred at room temperature for 2 h. The reaction mixture was quenched by the addition of saturated NH_4Cl (60 ml) and the aqueous layer was extracted twice with DCM. The combined organic phases were dried over $MgSO_4$ and the solvent was removed under reduced pressure. Silicagel flash column chromatography (10 % MeOH in DCM : DCM, 0 : 100 → 100 : 0, v/v) yielded the title ribonucleoside 2',3',5'-tri-*O*-benzoyl-[2-^{13}C, 1,3-$^{15}N_2$]-cytidine as a colourless foam (686.8 mg, 1.23 mmol, 69 %). ^{1}H-NMR (DMSO-d^6, 400 MHz): δ 8.36 (*s* br, 1 H, NH), 8.10 – 7.34 (*m,* 16 H, H6, Ph-H), 6.74 (*s* br, 1 H, NH), 6.17 (*m*, 1 H, H1'), 6.03 – 5.90 (*m*, 3H, H2', H3', H5), 4.82 – 4.66 (*m,* 3H, H4', H5'). ^{13}C-NMR ($CDCl_3$, 100 MHz): δ 166.2, 166.0, 166.0, 165.4, 165.3, 155.5, 141.5, 141.4, 137.9, 133.6, 133.4, 129.9, 129.8, 129.7, 129.4, 129.0, 128.8, 128.7, 128.6, 128.4, 128.2, 125.3, 96.0, 90.4, 79.8, 74.5, 71.1, 63.8. HRMS (ESI$^+$): calc. for $C_{29}{}^{13}CH_{25}{}^{15}N_2O_8$ $[M+H]^+$: 559.1689, found: 559.1690.

2.5 [2-^{13}C, 1,3-^{15}N$_2$]-Cytidine (3)

2',3',5'-Tri-*O*-benzoyl-[2-^{13}C, 1,3-^{15}N$_2$]-cytidine (655 mg, 1.17 mmol) was dissolved in anhydrous MeOH (25 ml). NaOMe (27 mg, 0.5 mmol) was added and the reaction mixture was stirred at room temperature for 5 h. Dowex H+ resin was added to the reaction mixture to decrease the pH to 7. The resin was removed using vacuum filtration and the filtrate concentrated under reduced pressure. The crude product was taken up in H_2O and aqueous phase was extracted twice with DCM. The aqueous layer was concentrated under reduced pressure to around 10 ml. The remaining solution was lyophilised to yield [2-^{13}C, *1,3*-^{15}N$_2$]-cytidine (**3**) as a colourless solid (280 mg, 1.14 mmol, 97%). ^{1}H-NMR (D_2O, 500 MHz): δ 7.85 (*dd*, *J* = 7.7 Hz, 6.0 Hz, 1 H, H6), 6.05 (*dd, J* = 7.7 Hz, 3.5 Hz, 1 H, H5), 5.90 – 5.89 (*m*, 1 H, H1'), 4.32 – 4.12 (*m,* 3 H, H2', H3', H4'), 3.94 – 3.79 (*m*, 2 H, H5'). ^{13}C-NMR (D_2O, 126 MHz): δ 165.9, 157.2, 141.6, 96.1, 90.3, 83.7, 73.9, 69.2, 60.7. HRMS (ESI$^+$): calc. for $C_8{}^{13}CH_{13}N^{15}N_2O_5$ [M+H]$^+$: 247.0902, found: 247.0901.

2.6 [2-^{13}C, 1,3-^{15}N$_2$]-2',3'-O-isopropylidene cytidine

[2-^{13}C, 1,3-^{15}N$_2$]-Cytidine (**3**) (180 mg, 0.73 mmol) was suspended in anhydrous acetone (15 ml). H_2SO_4 (100 µl) was added drop wise at 0 °C and the reaction mixture was stirred at room temperature overnight. The pH of the emulsion was adjusted to 9 by the addition of concentrated aqueous NH_4OH. The white precipitate was removed by vacuum filtration and the filtrate was concentrated under reduced pressure. Silicagel flash column chromatography (10 % MeOH in $CHCl_3$: $CHCl_3$, 0 : 100 → 10 : 90, v/v) yielded the title ribonucleoside [2-^{13}C, 1,3-^{15}N$_2$]-2',3'-*O*-isopropylidene cytidine as a colourless solid (182 mg, 0.636 mmol, 87 %). ^{1}H-NMR ($CDCl_3$, 400 MHz): δ 7.83 (*s* br, 1 H, NH), 7.36 – 7.33 (*m*, 1 H, H6), 6.57 (*s*, 1 H, NH), 5.85 (*dd*, *J* = 7.4 Hz, 3.6 Hz, 1 H, H5), 5.44 (*s* br, 1 H, H1'), 5.19 – 5.09 (*m*, 1 H), 5.02 – 5.00 (*m,* 1 H), 4.27 (*q*, *J* = 3.1 Hz, 1 H), 3.88 (*dd*, *J* = 12.3 Hz, 2.4 Hz, 1H, H5'), 3.77 (*dd*, *J* = 12.2 Hz, 3.3 Hz, 1H, H5'), 1.54 (*s*, 3 H, CH_3), 1.33 (*s*, 3 H, CH_3). ^{13}C-NMR ($CDCl_3$, 100 MHz): δ 166.5, 156.4, 144.4, 113.9, 98.0, 95.7, 87.8, 84.1, 80.7, 62.7, 27.4, 25.4. HRMS (ESI$^+$): calc. for $C_{11}{}^{13}CH_{17}N^{15}N_2O_5$ [M+H]$^+$: 287.1215, found: 287.1215.

2.7 5-Hydroxymethyl-[2-^{13}C, 1,3-^{15}N$_2$]-2',3'-O-isopropylidene cytidine

[2-^{13}C, 1,3-^{15}N$_2$]-2',3'-*O*-isopropylidene cytidine (129 mg, 0.45 mmol) was dissolved in aqueous KOH (0.5 M, 650 μl). Paraformaldehyde (23 mg, 1.5 eq) was added to the solution. The reaction mixture was heated (60 °C) using a microwave for 75 minutes. The reaction mixture was neutralized by the addition of dilute HCl and concentrated under reduced pressure. Silicagel flash column chromatography (20 % MeOH in $CHCl_3$: $CHCl_3$, 0 : 100 → 20 : 80, v/v) yielded the title compound 5-hydroxymethyl-[2-^{13}C,1,3-^{15}N$_2$]-2',3'-*O*-isopropylidene cytidine as a colourless solid (23 mg, 0.07 mmol, 16 %). ^{1}H-NMR (D_2O, 400 MHz): δ 7.80 (*d*, *J* = 6.2 Hz, 1 H, H6), 5.86 (*d*, *J* = 2.7 Hz, 1 H, H1'), 5.14 – 4.90 (*m,* 2 H, H2', H4'), 4.46 (*s*, 2 H, H7), 4.43 – 4.32 (*m*, 1 H, H3'), 3.82 (*dd*, *J* = 20.4 Hz, 4.8 Hz, 2 H, H5'), 1.62 (*s*, 3 H, C*H$_3$*), 1.42 (*s*, 3 H, C*H$_3$*). ^{13}C-NMR (D_2O, 100 MHz): δ 165.1, 156.7, 141.8, 114.6, 106.2, 93.6, 86.6, 84.4, 80.4, 61.4, 57.6, 26.0, 24.3. HRMS (ESI$^+$): calc. for $C_{12}{}^{13}CH_{19}N^{15}N_2O_6$ $[M+H]^+$: 317.1321, found: 317.1322.

2.8 5-Hydroxymethyl-[2-^{13}C, 1,3-^{15}N$_2$]-cytidine (4)

5-hydroxymethyl-[2-^{13}C,1,3-^{15}N$_2$]-2',3'-*O*-isopropylidene cytidine (23 mg, 0.07 mmol) was taken up in 90 % TFA and stirred at 0 °C for 1 h before ice cold Et_2O was added (2 ml). The precipitate was washed with ice cold Et_2O, taken up in water and lyophilised to obtain **4** as a colourless solid (19 mg, 0.069 mmol, 98 %). ^{1}H-NMR (D_2O, 500 MHz): δ 8.28 (*dd*, *J* = 7.5 Hz, 1.5 Hz, 1 H, H6), 5.89 (*s* br, 1 H, H1'), 4.50 (*s*, 2 H, H7), 4.34 – 4.15 (*m*, 3 H, H2', H3', H4'), 3.98 (*dd*, *J* = 13.0 Hz, 2.6 Hz, 1 H, H5'), 3.83 (*dd*, *J* = 13.0 Hz, 3.6 Hz, 1 H, H5'). ^{13}C-NMR (D_2O, 126 MHz): δ 158.1, 147.8, 142.9, 105.8, 90.3, 84.1, 74.1, 68.6, 59.9, 56.7. HRMS (ESI$^+$): calc. for $C_9{}^{13}CH_{15}N^{15}N_2O_6$ $[M+H]^+$: 277.1008, found: 277.1006.

2.9 5-[Methyl-D$_3$]-[6-D]-cytidine (6)

5-[Methyl-D$_3$]-[6-D]-cytidine (***6***) (618 mg, 2.37 mmol, 44 %) was obtained from thymine-D$_4$ (700 mg, 5.38 mmol) using the same sequence of reactions as described in section 2.2 – 2.4. ^{1}H-NMR (D_2O, 500 MHz): δ 5.89 (*d*, *J* = 4.1 Hz, 1H, H1'), 4.31 – 4.21 (*m*, 2 H, H2', H3'), 4.13 – 4.10 (*m*, 1 H, H4'), 3.93 (*dd*, *J* = 12.8 Hz, 2.9 Hz, 1 H, H5'), 3.82 (*dd*, *J* = 12.8 Hz, 4.2 Hz, 1 H, H5'). ^{13}C-NMR (D_2O, 126 MHz): δ 180.3, 165.2, 156.4, 104.2, 89.9, 83.8, 73.6, 69.1, 60.5. HRMS (ESI$^+$): calc. for $C_{10}CH_{11}D_4N_3O_5$ $[M+H]^+$: 262.1336, found: 262.1340.

3. Isolation of total RNA, purification and polyA pull-down

HEK293T cells were cultured in Dulbecco's Modified Eagle Medium (*Life Technologies*) supplemented with 10 % fetal bovine serum at 37 °C in 5 % CO_2 atmosphere. The cells were isolated for RNA extraction at 75 % confluence level by trypsinisation followed by centrifugation. The resulting cell pellet was washed twice with PBS before RNA isolation was continued. E. coli (DH5α) cells were grown in LB medium to log phase at 37 °C.

Total RNA extraction from cells and tissues was performed by direct addition of the TRI-reagent (*Sigma*) following the manufacturer's protocol.

RNA was purified using RNA Clean & Concentrator kit (*Zymo Research*) before any downstream applications. PolyA RNAs were isolated by subjecting total RNA to a double round of polyA selcection using the Oligotex mRNA kit (*QIAGEN*) according to the manufacturer's protocol.

4. RNA digestion

Digestion enzyme master mix was prepared by combining benzonase (250 U/µl, 0.625 µl, *Sigma Aldrich*), phosphodiesterase I from Crotalus adamanteus venom (10 mU/µl, 10 µl, *Sigma Aldrich*) and Antarctic phosphatase (5 U/µl, 20 µl, *New England Biolabs*). Aqueous solutions of total RNA (1 µg in 13.25 µl final volume) were mixed with 5x digestion buffer (5 µl, Tris-HCl pH 8 (20 mM), $MgCl_2$ (20 mM), NaCl (100 mM)) and digestion enzyme stock solution (0.5 µl) and water (6.25 µL) or, for SID quantitation purposes, a solution of stable isotope labeled nucleosides (100 nM, 6.25 µl, 25 nM final concentration), followed by incubation at 37 °C for 14 h. The nucleoside mixture was subsequently cleaned-up by filtration over Amicon Ultra 0.5 ml (10 kDa MWCO, Merck-Milipore) spin columns.

5. LC-MS/MS analysis

5.1 LC-MS/MS analysis of labeled murine tissues

High resolution LC-MS/MS experiments to identify m^5C, hm^5C and f^5C and the isotopologues m^5C + 4 SIL, hm^5C + 3 SIL and f^5C + 2 SIL in labeled murine tissues were performed using a Dionex Ultimate 3000 RSLC nanoHPLC system coupled to a Thermo Scientific Q-exactive tandem mass spectrometer. LC conditions typically consisted of a 10 – 95 % multistep gradient using H_2O / MeCN (0.1% formic acid) as the mobile phase. Separation was achieved using a manually packed Hypercarb (Dr Maisch, 3µm particle size) fused silica capillary column (l = 20 mm, id = 75 µm) at 1500 nL / min flow rate. The Q-exactive quadrupole-orbitrap hybrid tandem MS spectrometer was

fitted with a nanospray probe (1 KV) and preset to select for the individual target ions. The NCE was set to 10 and the resolution was 70000 FHWM at 400 m/z. Data (Table S6.4) was processed using the Thermo Scientific Xcalibur software package V 2.2.44.

Determination of the isotope incorporation by integration (Figure S6.1, Table S6.3) of the XIC's for m^5C, hm^5C and f^5C and the isotopologues m^5C + 4 SIL, hm^5C + 3 SIL and f^5C was performed as described in 5.2.

5.2 Quantitative LC-MS/MS analysis:

LC-MS/MS analysis was carried out using an Agilent 1290 Infinity UHPLC and autosampler coupled to an AB Sciex 6500 Triple Quadrupole mass spectrometer using 5 minute run time cycles.

LC was performed on a Waters Acquity UPLC® HSS T3 column (100 x 2.1 mm, 1.8 µm particle size) kept at 50°C, applying a gradient starting at 100% of 0.1% formic acid in water followed by increasing proportions of 0.1% formic acid in acetonitrile up to 15%, at a flow rate of 300 µL/min over 3 minutes. An additional 2 minutes were used to wash and re-equilibrate the column under the starting conditions.

The MS was operated using positive electrospray ionisation at 4750V with a source temperature of 500°C. Multiple reaction monitoring (MRM) was used with dwell times of 20 msec to measure cytidine (C), 5-methylcytidine (m^5C), 5-hydroxymethylcytidine (hm^5C), 5-formylcytidine (f^5C) and their corresponding stable isotope labels (SIL) versions. A 'detuned' cytidine transition (using a lower abundance fragment ion) was also monitored due to detector saturation from high levels of cytidine in samples. Transitions used: **244.1→112.0** (C), **244.1→69.2** (C detuned), **247.1→115.0** (C+3 SIL), **258.0→126.1** (m^5C), **262.0→130.1** (m^5C + 4 SIL), **274.0→142.1** (hm^5C), **277.0→145.1** (hm^5C + 3 SIL), **271.9→140.1** (f^5C) and **273.9→142.1** (f^5C + 2 SIL).

Data was processed using AB Sciex Multiquant (Ver. 2.1.1) and, where required, concentrations were back calculated from calibration lines produced from authentic reference standards over the ranges of 5-15,000 nM (C), 0.025-750 nM (m^5C) and 0.005-150 nM (hm^5C and f^5C).

6. Tables and Figures:

	Sample	BR 1			BR 2		
		TR 1	TR 2	TR 3	TR 1	TR 2	TR 3
hm^5C (% of total C)	E. coli	0.00042	0.00036	0.00075			
	M. Jannaschii	0.00061	0.00071	0.00065	0.00037	0.00037	
	C. elegans	0.00027	0.00029	0.00009	0.00026	0.00012	0.00024
	A. thaliana		0.00478	0.00450	0.00521	0.00473	0.00503
	HEK293T	0.00032	0.00035	0.00034	0.00033	0.00033	0.00031
	Mouse Kidney	0.00164	0.00182	0.00165	0.00187	0.00197	0.00202
	Mouse Cerebellum	0.00162	0.00133	0.00150	0.00168	0.00155	0.00153
	Mouse Cerebrum	0.00146	0.00134	0.00124	0.00122	0.00117	0.00143
	Mouse Spleen	0.00114	0.00116	0.00096	0.00203	0.00212	0.00182
	Mouse Liver	0.00055	0.00066	0.00081	0.00127	0.00128	0.00141
	Mouse Testis	0.00173	0.00158	0.00178	0.00120	0.00114	0.00116
	Mouse Heart	0.00264	0.00256	0.00244	0.00204	0.00183	0.00188
	Mouse Lung	0.00142	0.00180	0.00179	0.00119	0.00110	0.00128

Table S6.1: *The levels of hm^5C in total RNA samples isolated from different model organisms and various male mouse tissues from two biological replicates (BR) and three technical replicates (TR).*

	Sample	BR 1			BR 2		
		TR 1	TR 2	TR 3	TR 1	TR 2	TR 3
m^5C (% of total C)	E. coli	0.00637	0.00609	0.00763	0.00423	0.00459	0.00395
	M. Jannaschii	1.62125	1.53282	1.55900	1.59015	1.59009	1.63167
	C. elegans	0.36120	0.34747	0.32746	0.33028	0.33614	0.33740
	A. thaliana		0.36323	0.37755	0.38231	0.39752	0.40294
	HEK293T	0.36648	0.36475	0.38087	0.39576	0.41165	0.41401
	Mouse Kidney	0.39586	0.39824	0.38617	0.77872	0.78544	0.74622
	Mouse Cerebellum	0.78250	0.81687	0.77354	0.87480	0.87864	0.80371
	Mouse Cerebrum	0.77021	0.69093	0.72927	0.72927	0.68076	0.75697
	Mouse Spleen	0.53639	0.55596	0.53732	0.79546	0.83646	0.78055
	Mouse Liver	0.50174	0.47429	0.47077	0.87220	0.80035	0.80269
	Mouse Testis	0.85402	0.82171	0.84292	0.75817	0.75663	0.78089
	Mouse Heart	1.28463	1.15282	1.13240	0.85163	0.83379	0.89523
	Mouse Lung	0.77398	0.79603	0.79922	0.54384	0.53919	0.51416

Table S6.2: *The levels of m^5C in total RNA samples isolated from different model organisms and various male mouse tissues from two biological replicates (BR) and three technical replicates (TR).*

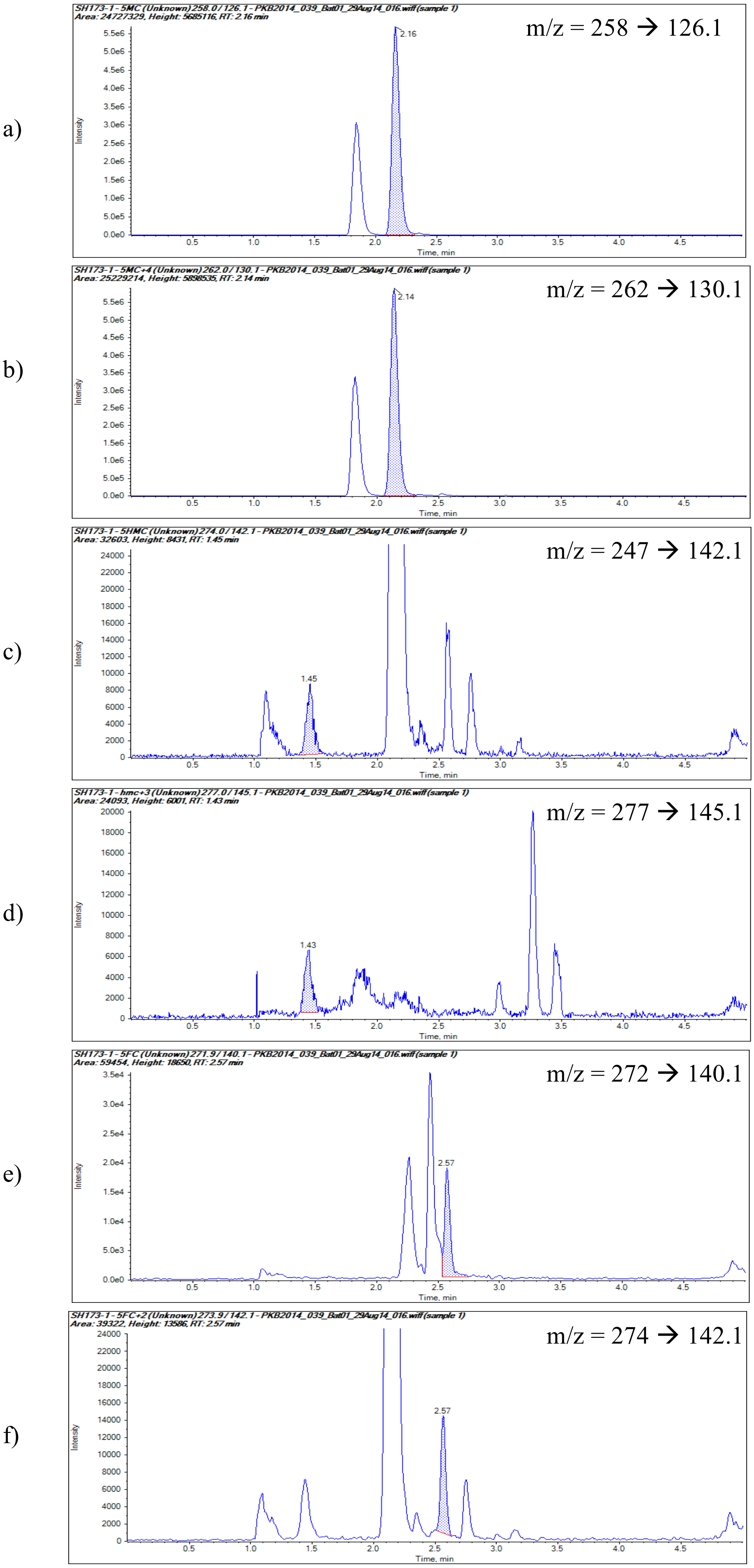

Figure S6.1: *Extracted ion count (XIC) for m^{5}C + 4 Da, hm^{5}C + 3 Da, f^{5}C + 2 Da and their unlabeled analogues.*

A		m5C	m^5C + 4 Da	hm^5C	hm^5C + 3 Da	f^5C	f^5C + 2 Da
Brain Female	1	15559103	15696272	23840	16126	42823	25619
	2	24727329	25229214	32603	24093	59454	39322
Brain Male	1	36400290	15687630	14392	14152	25954	13765
	2	15382648	24919005	24222	40344	33011	38093

B		m^5C : m^5C + 4 Da	hm^5C : hm^5C + 3 Da	f^5C : f^5C + 2 Da
Brain Female	1	50 : 50	60 : 40	63 : 37
	2	49 : 51	58 : 42	60 : 40
Brain Male	1	38 : 62	50 : 50	65 : 35
	2	38 : 62	38 : 62	56 : 54

Table S6.3: *A) Absolute XIC values for nucleosides measured in the SIL-mouse tissues B) Relative distribution of mouse tissue nucleosides derived from A.*

**Base**	**Source**	**Isotopes**	**Formula**	**Calculated $[M+H]^+$**	**Found $[M+H]^+$**	**Δ (ppm)**
C	natural	-	$C_4H_6N_3O$	112.05054	112.05098	3.9
m^5C	natural	-	$C_4H_8N_3O$	126.06619	126.06647	2.2
	Met*	$^{13}C, D_3$	$C_4{}^{13}CH_5D_3N_3O$	130.08837	130.08861	1.8
hm^5C	natural	-	$C_5H_8N_3O_2$	142.06110	142.06139	2.0
	Met*	$^{13}C, D_2$	$C_4{}^{13}CH_6D_2N_3O_2$	145.07701	145.07715	1.0
f^5C	natural	-	$C_5H_6N_3O_2$	140.04545	140.04553	0.6
	Met*	^{13}C, D	$C_4{}^{13}CH_5DN_3O_2$	142.05508	142.05530	1.5

Table S6.4: *Accurate masses of nucleobases detected in RNA digests from SIL-mouse tissues.*

	m^5C (% of total C)	hm^5C (% of total C)	(% m^5C) / (% hm^5C)
HEK293T polyA 1	0.09517	0.00369	26
HEK293T polyA 2	0.02538	0.00103	25
HEK293T total RNA	0.39576	0.00033	1199

Table S6.5: *The levels of m^5C and hm^5C in polyA and total RNA samples isolated from HEK293T cells.*

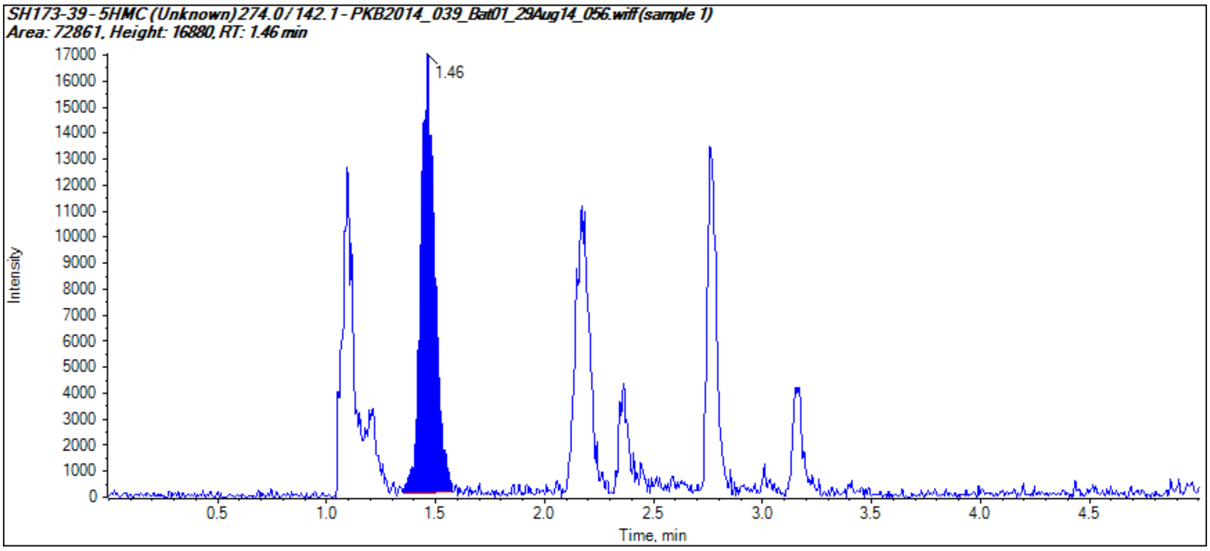

Figure S6.2: *Extracted ion count (XIC) for hm^{5}C in polyA RNA from HEK293T cells.*

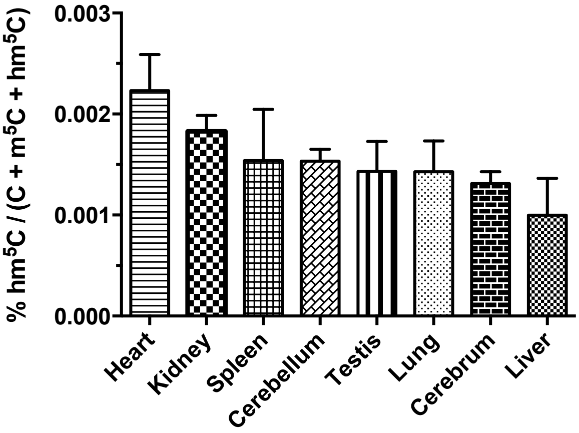

Figure S6.3: *hm^{5}C abundance measured in murine tissues, relative to the sum of rC-nucleosides.*

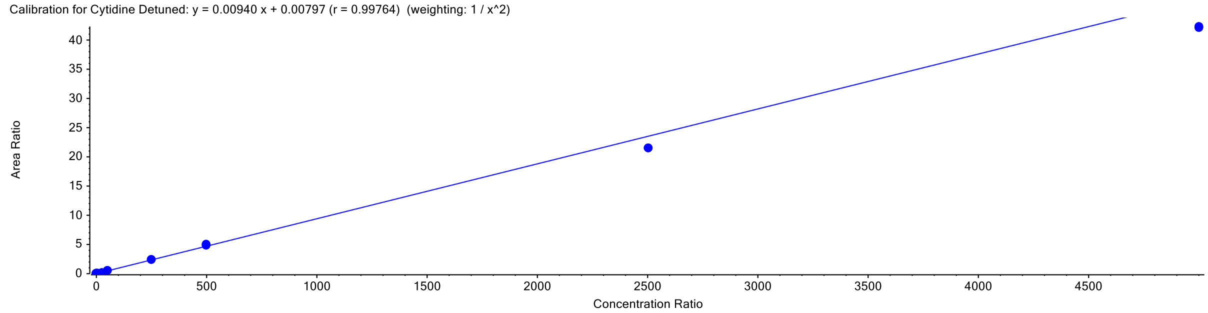

Figure S6.4a: *Representative calibration curve for C-detuned.*

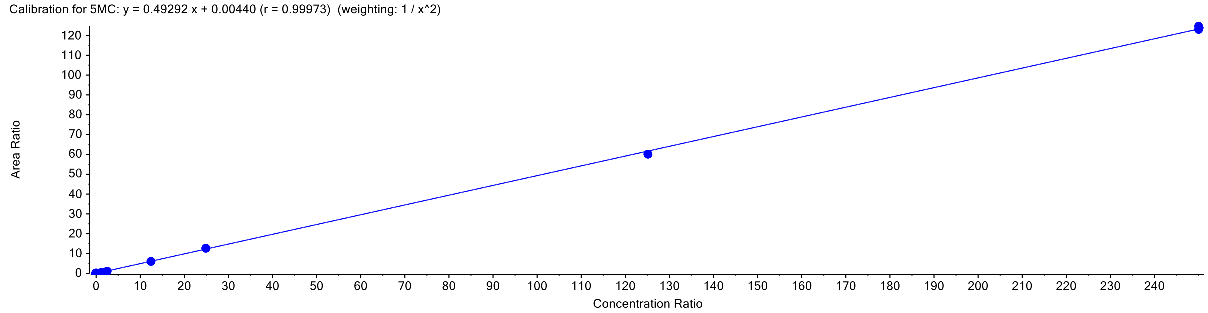

Figure S6.4b: *Representative calibration curve for m^{5}C.*

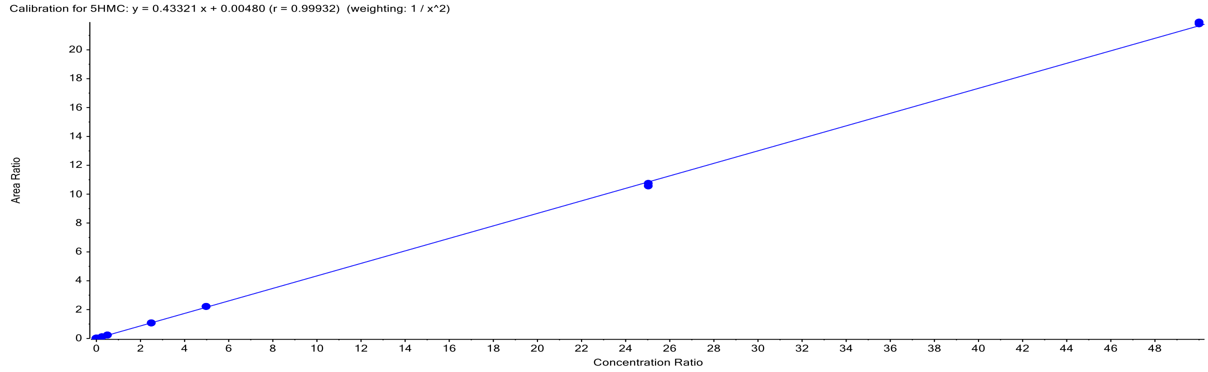

Figure S6.4c: *Representative calibration curve for hm^{5}C.*

7. NMR spectra of SILs

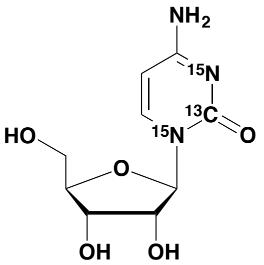

7.1 [2-^{13}C, 1,3-^{15}N$_2$]-Cytidine (3)

^{1}H-NMR (500 MHz, D_2O):

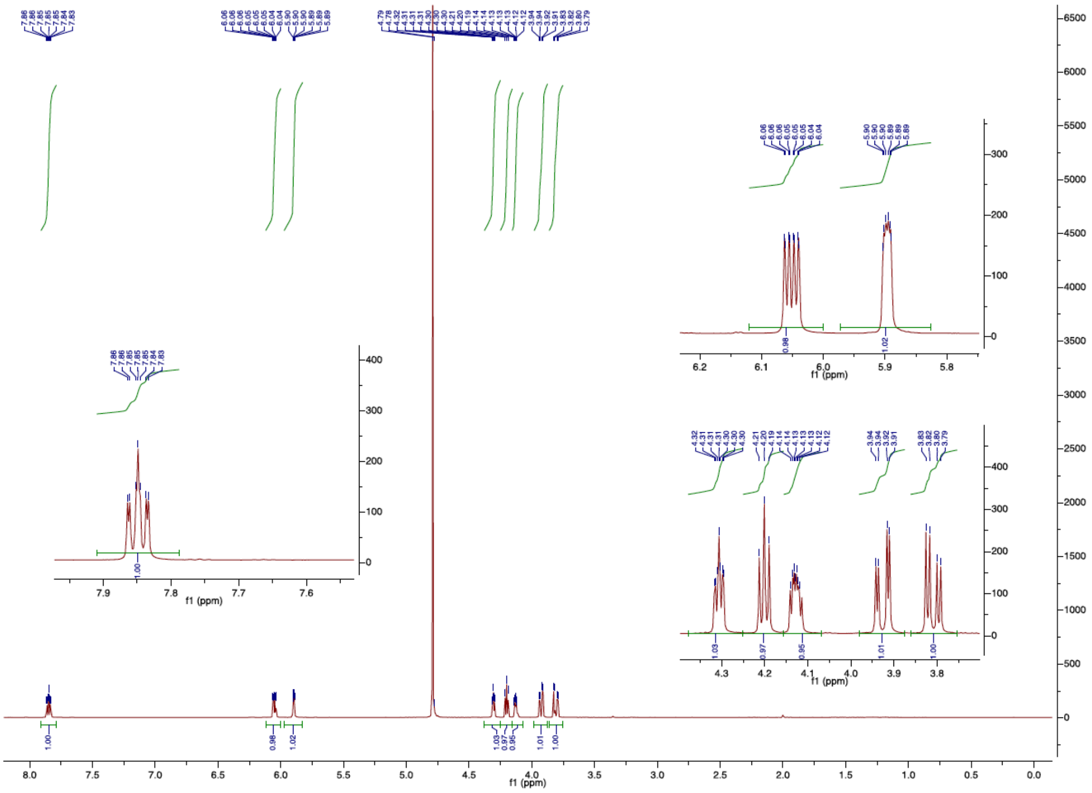

^{13}C-NMR (126 MHz, D_2O):

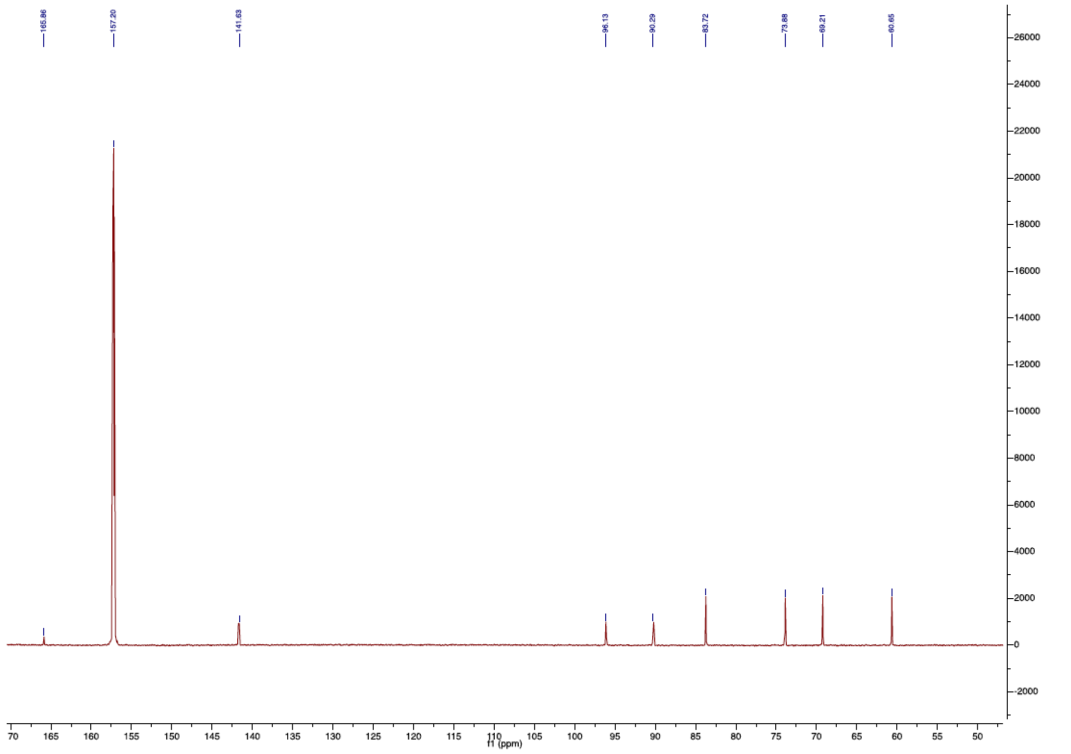

7.2 5-[Methyl-D3]-[6-D]-cytidine (6)

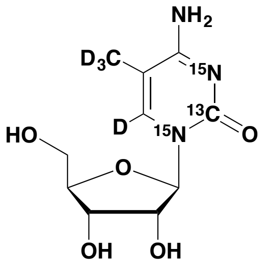

^{1}H-NMR (500 MHz, D_2O):

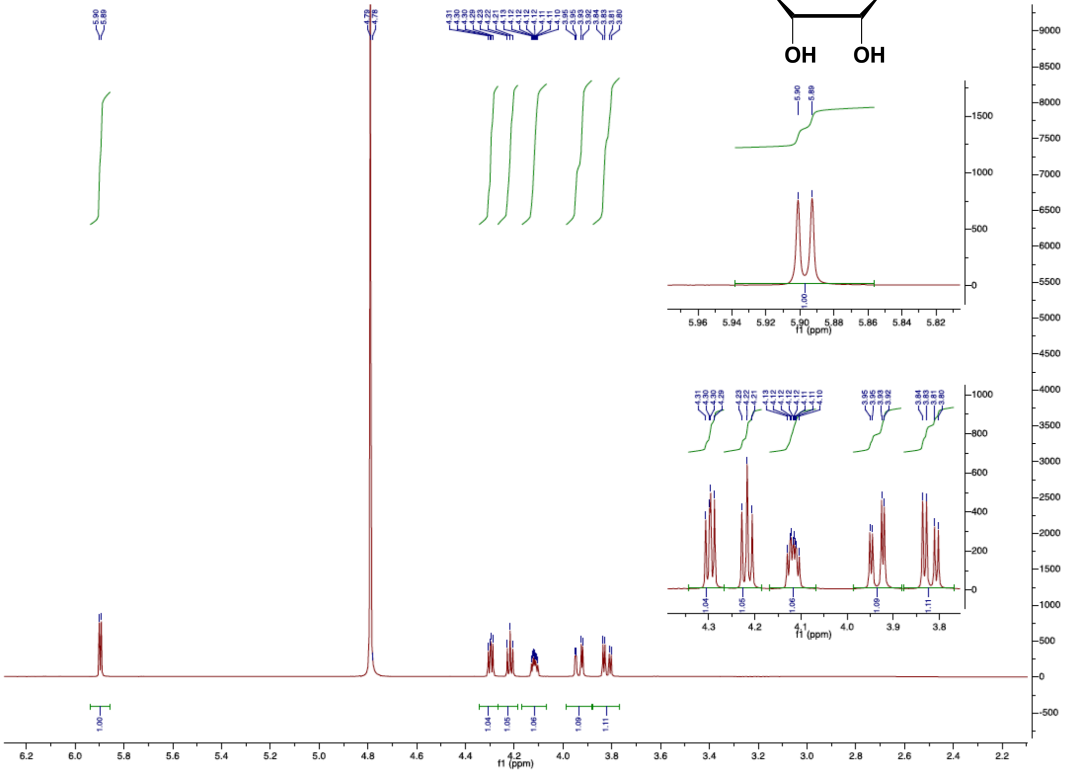

^{13}C-NMR (126 MHz, D_2O):

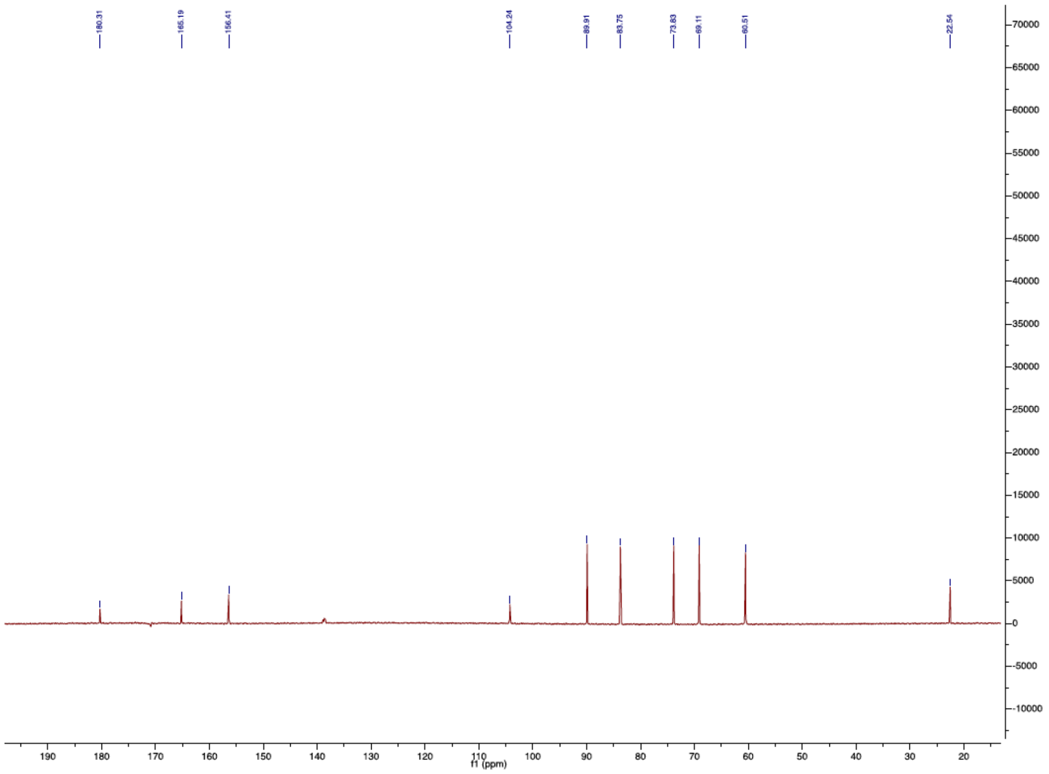

7.3 5-Hydroxymethyl-[2-^{13}C, 1,3-^{15}N$_2$]-cytidine (4)

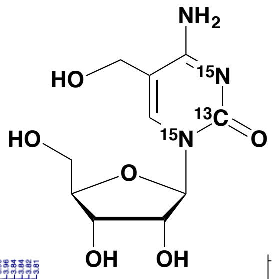

^{1}H-NMR (500 MHz, D_2O):

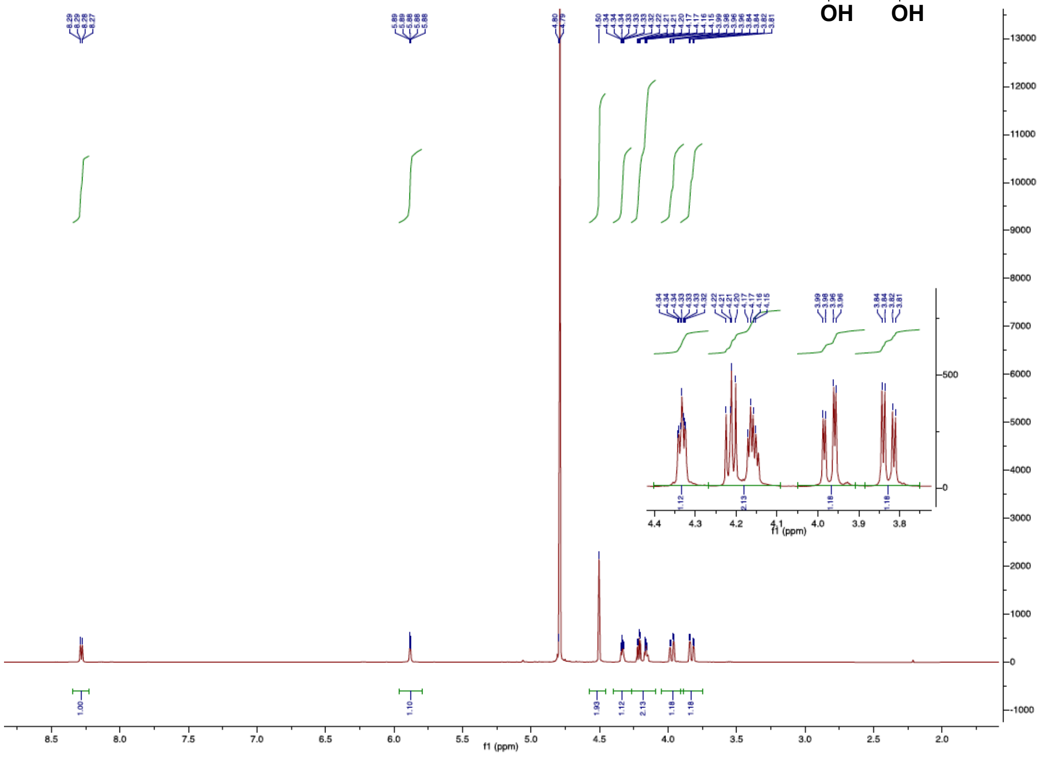

^{13}C-NMR (126 MHz, D_2O):

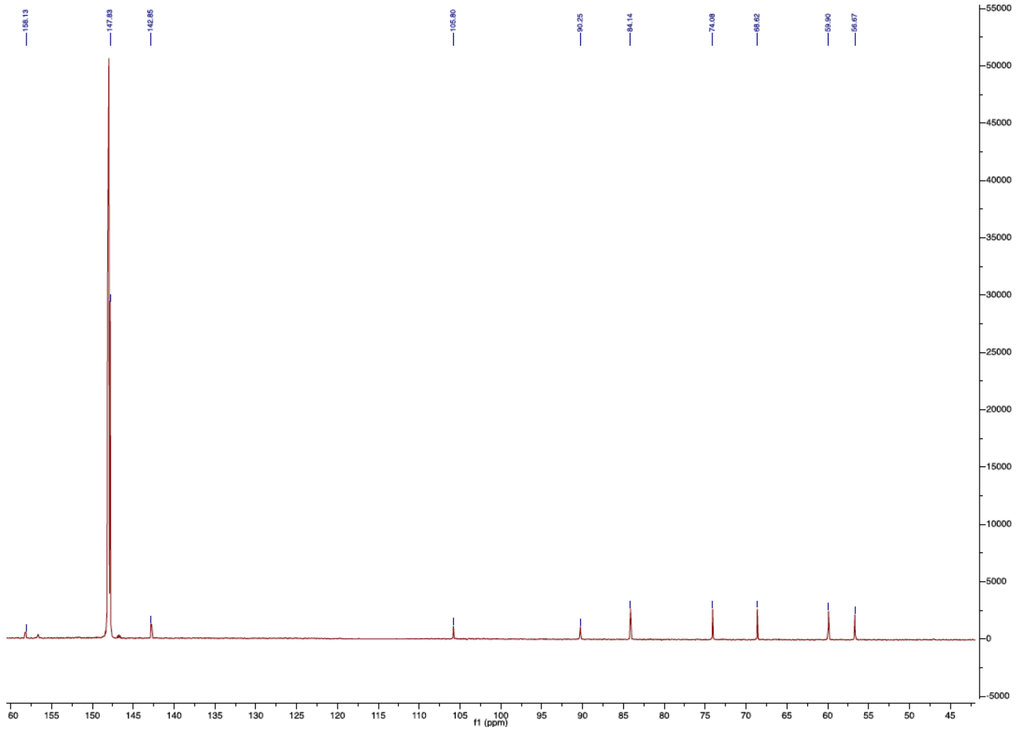

Supplement: Supplementary file 1 [file cbic0016-0752-sd1.pdf]
